# Rare variation of the right internal jugular vein: a case study

**DOI:** 10.1590/1677-5449.007018

**Published:** 2018

**Authors:** Camila Gomes de Souza, Lucas Piraciaba Cassiano Dias, Rafael Vargas, Luiz Alberto Diniz do Nascimento, Mônica Volino-Souza, Gustavo Vieira de Oliveira

**Affiliations:** 1 Universidade Federal do Rio de Janeiro – UFRJ, Departamento de Medicina, Macaé, RJ, Brasil.

**Keywords:** anatomy, surgical procedures, blood vessels, jugular veins, anatomia, procedimentos cirúrgicos, vasos sanguíneos, veias jugulares

## Abstract

This study reports on a rare variation of the right internal jugular vein (IJV) identified during routine anatomic dissection of a male cadaver. The right IJV had a tributary located parallel and medially to the IJV itself. This branch of the IJV emerged between the transverse processes of the 3rd and 4th cervical vertebrae and drained into the junction between the right internal jugular and brachiocephalic veins. The present study described a rare branch of the right IJV, which is important knowledge for surgeons, in order to prevent accidental injury and bleeding during surgical procedures.

## Resumo

O presente estudo demonstrou uma variação rara da veia jugular interna direita (VJI), identificada durante uma dissecção de rotina de um cadáver masculino. A VJI direita apresentou uma tributária localizada paralelamente e medialmente à referida veia. Essa tributária da VJI emergiu entre os processos transversos das 3ª e 4ª vértebras cervicais e drenou para a junção entre as veias jugular interna e braquiocefálica direita. O conhecimento da variação anatômica demonstrado no presente estudo é importante para os cirurgiões, a fim de prevenir lesões acidentais e sangramento durante procedimentos cirúrgicos.

## INTRODUCTION

 The internal jugular vein (IJV) is responsible for most of the venous drainage of cranial cavity structures and deep portions of the face and neck. The IJV originates at the base of the skull in the posterior compartment of the jugular foramen. Subsequently, the IJV runs down in the vertical direction, on the side of the neck, within the carotid sheath. At the level of the sternal extremity of the clavicle, the right IJV unites with the ipsilateral subclavian vein to form the brachiocephalic vein. During its course, the IJV is, at first, located laterally to the internal carotid artery and then to the common carotid artery. 1

 Knowledge of the IJV is important, since it is routinely used to obtain central venous access for several purposes, such as blood sampling, administration of antibiotics and chemotherapy drugs, for vascular access for hemodialysis, and to monitor right atrial pressure. The IJV is also required for insertion of transjugular intrahepatic portosystemic shunts, to conduct transjugular liver biopsies, and for placement of inferior vena cava filters. Furthermore, cervical and neck dissection is a common procedure in management of head and neck cancer. Thus, success of surgical management relies on adequately recognizing patient-specific anatomic structures, to avoid complications such as hemorrhage, nerve damage, and chylous fistulae. 2 ^,^ 3 In this context, this case study presents a rare IJV anatomical variation that may contribute to cervical therapeutic and diagnostic procedures. 

## CASE REPORT

**Figure 1:**
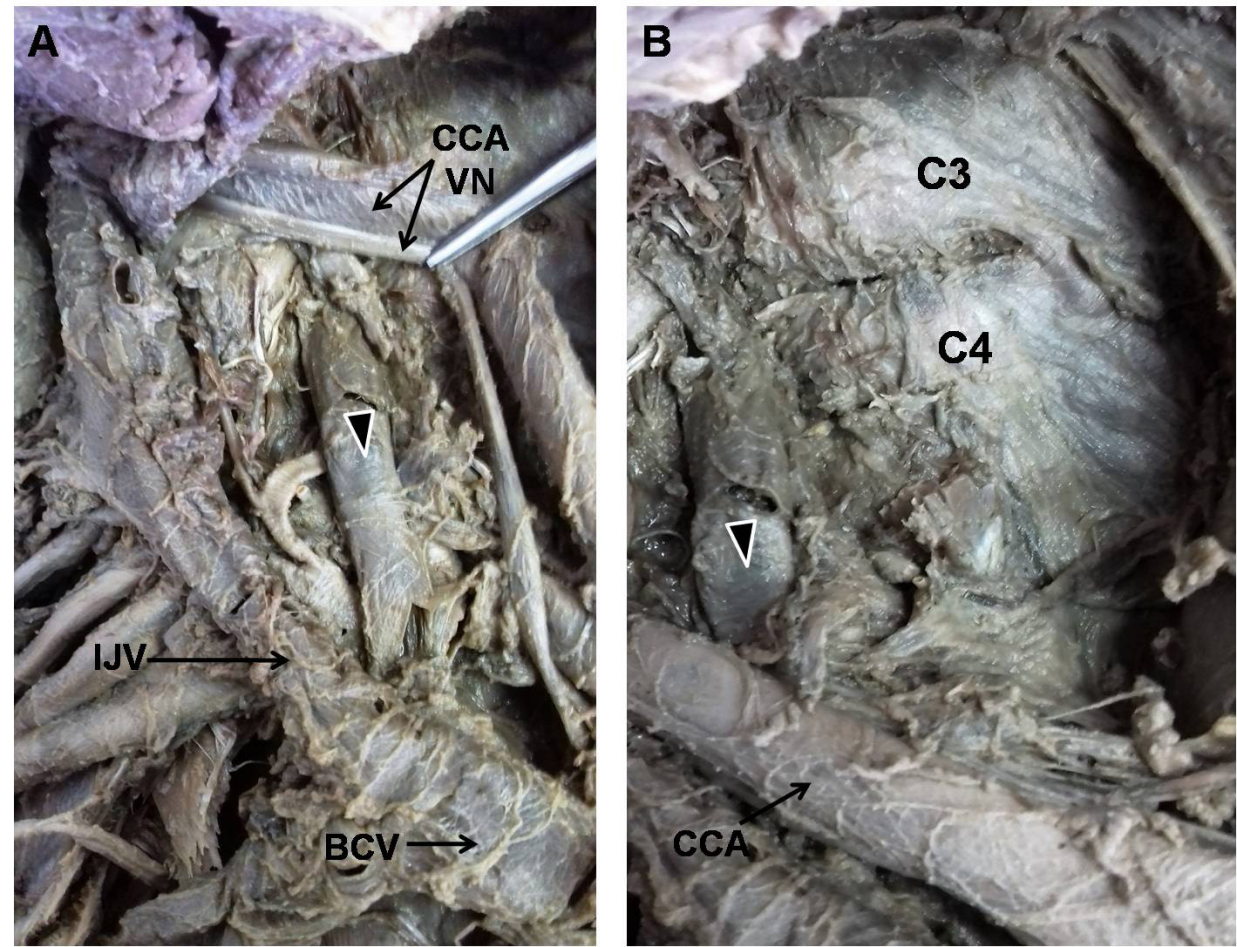
Right IJV with its parallel and medial branch (A). This branch of the IJV emerged between transverse processes of the 3rd and 4th cervical vertebrae and drained into the junction between the right internal jugular and brachiocephalic veins (B). BCV = brachiocephalic vein; C3 and C4 = 3rd and 4th cervical vertebrae; CCA = common carotid artery; IJV = internal jugular vein; VN = vagus nerve. Black arrow, branch of the IJV.

 During routine anatomic dissection of an 83-year-old male cadaver, a variation of the right IJV was observed. The right IJV exhibited a tributary located parallel and medially to the IJV itself. This branch of the IJV emerged between the transverse processes of the third and fourth cervical vertebrae and drained into the junction between the right internal jugular and brachiocephalic veins. The branch was 6.5 cm in length, running from the transverse processes of the cervical vertebrae to the junction between the internal jugular and brachiocephalic veins. It ran down dorsally in relation to the vagus nerve and common carotid artery ( Figure 1 ). 

## DISCUSSION

 This study presents a rare IJV branch located in parallel and medially to the IJV itself. The IJV is the largest caliber blood vessel that runs down the side of the neck in a vertical direction. It serves as a major surgical landmark for adjacent structures during neck dissection. Furthermore, the right IJV is preferred to the left for cannulation procedures, to avoid injury to the thoracic duct, which is on the left, because it offers a more direct route to the superior vena cava, and because of the low level of the pleural dome. 4 Therefore, due to the clinical implications of right IJV anatomy, several studies have reported different anatomical variations of this vessel. 5 ^-^ 7

 Micozkadioglu and Erkan 6 reported a lateral branch found at the lower level of the right IJV running to the lateral side of the neck. Deepak et al. 5 demonstrated two cases of IJV anatomical variation: (i) the presence of a IJV trifurcation at the thyroid cartilage level; and (ii) a posterior IJV branch entering deep into the anterior border of the trapezius muscle. Shetty et al. 7 reported that the facial, external jugular, and suprascapular veins of the right side of the neck joined to form a large vein, which drained into the junction between the right internal jugular and subclavian veins. 

 Additionally, studies have reported other IJV anatomical variations, including duplications and fenestrations. 8 IJV duplication refers to branches of the vein that remain separate throughout their entire lengths, draining separately into the subclavian vein, whilst IJV fenestration refers to a bifurcation that reunites the proximal and the subclavian vein. Both IJV duplication and fenestration have been well described. 

 The IJV comprises one of two major intracranial blood pathways and, because of its uniform and superficial position, emergency physicians, cardiologists, oncologists and nephrologists routinely use this vessel for central venous access and for estimating central venous pressure. Adequate knowledge of the IJV’s position and its variations is also critical for cervical lymph node clearance performed by oncology surgeons and in most cervical operations. Furthermore, the IJV is a landmark for radiologists, especially when interpreting computed tomography angiography. Thus, IJV anatomical variations may be obstacles to various clinical procedures, resulting in injury to the vasculature and spinal accessory nerve during neck dissection, failure to remove all cancerous tissues, and incorrect neck pathology diagnoses. 9

 From an embryological point of view, the anterior cardinal vein, also known as the precardinal vein, drains the cephalic regions of the embryo. The right anterior cardinal, common cardinal and posterior cardinal veins undergo a major evolutionary process to become the superior vena cava and its tributaries. The distal segments of the bilateral anterior cardinal veins become the bilateral IJVs, which drain the head and neck. An anastomosis grows from the left anterior cardinal vein to the right anterior cardinal vein to form the left brachiocephalic vein. Hence, the blood from the left IJV goes through the left brachiocephalic vein, draining directly into the superior vena cava. 10 Due to the complicated nature of development of the important veins of the right side (the superior vena cava and its tributaries), there are many opportunities for abnormal development, regression or anastomosis, generating anatomical variations. 

## CONCLUSION

 The present study describes a rare branch of the right IJV, which is important knowledge for surgeons, in order to prevent accidental injury and bleeding during surgical procedures. Furthermore, adequate knowledge of IJV anatomical variations will also aid in avoiding radiologic misinterpretations or misidentifications of neck veins during conventional radiographic procedures, such as angiography. 
